# Metal coordination in C_2_N-like materials towards dual atom catalysts for oxygen reduction†

**DOI:** 10.1039/d1ta09560a

**Published:** 2022-02-11

**Authors:** Jesús Barrio, Angus Pedersen, Jingyu Feng, Saurav Ch. Sarma, Mengnan Wang, Alain Y. Li, Hossein Yadegari, Hui Luo, Mary P. Ryan, Maria-Magdalena Titirici, Ifan. E. L. Stephens

**Affiliations:** Department of Materials, Royal School of Mines, Imperial College London London SW27 AZ England UK i.stephens@imperial.ac.uk; Department of Chemical Engineering, Imperial College London London SW7 2AZ England UK m.titirici@imperial.ac.uk; Advanced Institute for Materials Research (WPI-AIMR), Tohoku University 2-1-1 Katahira, Aobaku Sendai Miyagi 980-8577 Japan

## Abstract

Single-atom catalysts, in particular the Fe–N–C family of materials, have emerged as a promising alternative to platinum group metals in fuel cells as catalysts for the oxygen reduction reaction. Numerous theoretical studies have suggested that dual atom catalysts can appreciably accelerate catalytic reactions; nevertheless, the synthesis of these materials is highly challenging owing to metal atom clustering and aggregation into nanoparticles during high temperature synthesis treatment. In this work, dual metal atom catalysts are prepared by controlled post synthetic metal-coordination in a C_2_N-like material. The configuration of the active sites was confirmed by means of X-ray adsorption spectroscopy and scanning transmission electron microscopy. During oxygen reduction, the catalyst exhibited an activity of 2.4 ± 0.3 A g_carbon_^−1^ at 0.8 V *versus* a reversible hydrogen electrode in acidic media, comparable to the most active in the literature. This work provides a novel approach for the targeted synthesis of catalysts containing dual metal sites in electrocatalysis.

## Abstract 2

Single-atom catalysts, in particular the Fe–N–C family of materials, have emerged as a promising alternative to platinum group metals in fuel cells as catalysts for the oxygen reduction reaction.

## Graphical abstract

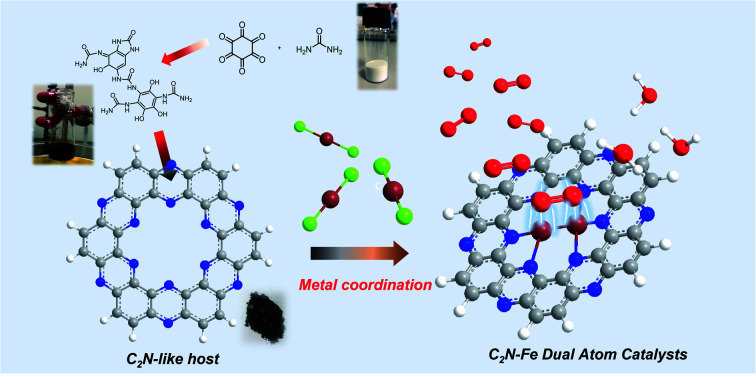

## Introduction

Nitrogen doped coordinated single metal atom catalysts have attracted widespread attention in the field of electrochemistry owing to their well-defined active sites, high metal utilisation, and catalytic activity.^1–5^ In particular, iron–nitrogen–carbon catalysts have emerged as a potentially more sustainable alternative to platinum group metals (PGMs) in the oxygen reduction reaction (ORR),^6,7^ the largest cause of overpotential in fuel cells. Since the initial report by Jasinski using phthalocyanines as a model molecular system,^8^ significant research efforts have been dedicated to enhancing the density of active sites or the intrinsic activity of the active site.^9–11^ Nevertheless, even the most active single metal atom catalysts still require a significant overpotential of >0.4 V to reach a substantial turnover frequency of 1.5 × 10^−1^ site s^−1^ (0.8 V,^12^ CO chemisorption); the reason for this overpotential is because of suboptimal scaling relations between the binding energies of the intermediates, in particular between *OOH and *OH.^13,14^ Density functional theory (DFT) simulations suggest that a dual atom catalyst, with two metals at the active site, surrounded by more inert elements (Au, C, N, S, Hg, *etc.*) could yield more optimal scaling relations between *OOH and *OH.^14–17^ As such, dual metal atom catalysts should in principle require negligible overpotential to achieve high current densities, or orders of magnitude higher activity at a given overpotential. These materials are inspired by the structure of enzymes active sites, which display two earth-abundant metals, such as Fe, in atomic proximity, such as the cytochrome *c* oxidase.^18–20^ While our theoretical understanding of the class of this materials has advanced enormously,^21–25^ experimental progress has been more limited. In particular, most routes for the synthesis of these bio-inspired catalysts containing Fe entail high temperatures, which result in the formation of undesirable iron carbides, iron nitrides and – under carbothermal reduction – iron nanoparticles.^11,26^ Researchers have proposed several routes to synthesise dual metal atom catalysts including most commonly; deposition (atomic layer,^27^ chemical vapour,^28^ and electrochemical^29^) and high temperature pyrolysis of encapsulated metal precursors within metal organic frameworks,^30–32^ or simply with multiple components.^33–35^ For pyrolysis-based approaches, encapsulation of the metal precursor may prevent undesirable side reactions; however, complete encapsulation of the metal will prevent access to reactants, limiting their use as catalysts. We posit that the controlled loading and stabilisation of dual atoms seems most reasonably achieved post-pyrolysis at ambient temperatures in a suitable pore or framework.

Consequently, here we take a two-step approach, where (i) a metal-free support with high nitrogen content is prepared in the presence of a porogen (such as MgCl_2_·6H_2_O) and subsequently (ii) Fe is coordinated in the N-rich pores. Fellinger and co-workers pioneered this method for single atom Fe–N_4_ ORR catalysts.^36^ We envision that by employing this technique on a support with appropriate porosity will result in the stabilisation of dual atom catalysts, avoiding their aggregation.^37^ We have chosen to host the metal centres in C_2_N-like covalent organic frameworks. The nitrogen lined pores in this novel class of 2D porous materials, as well as its pore size of 8.3 Å makes it an ideal candidate for hosting dual catalysts.^38,39^ Furthermore, DFT calculations suggest high diffusion barriers for dual metal atoms in a C_2_N framework, meaning that there would be a lower propensity to form aggregates in these materials.^21,40^ Therefore, in this work we construct dual metal atom catalysts by controlled metal coordination in a C_2_N-like material, which is prepared by pyrolysis at high temperatures of a cross-linked precursor.^41^ The structure of the as-synthesised material was elucidated by means of X-ray absorption spectroscopy, electron paramagnetic resonance spectroscopy, and scanning transmission electron microscopy. The performance of the catalysts for the ORR was evaluated in acidic media, using rotating disk electrode measurements.

## Experimental

### Synthetic procedures

C_2_N-Like materials were prepared as reported in literature with slight modifications.^41^ Namely, hexaketocyclohexane octahydrate (97% Sigma Aldrich, 936 mg, 3 mmol) and urea (99.5% GF Healthcare life Science, 837 mg, 13.5 mmol) were mechanically mixed by means of a pestle and mortar and subsequently placed in a glass vial. A cross-linked complex was formed by gently heating the vial at 90 °C. The cross-linked complex was ground with magnesium chloride hexahydrate (99% Sigma Aldrich) in a weight ratio of 1 : 8, and the mixture pyrolysed in a ceramic crucible at 700–1000 °C for one hour under N_2_ atmosphere (500 mL min^−1^) with 3 °C min^−1^ heating rate. The materials, labelled as C_2_N *X* (where *X* denotes the pyrolysis temperature), were collected and washed with 2 M HCl (prepared by dilution of HCl fuming 37%, Merck) overnight to remove the remaining MgCl_2_ or MgO species. The catalyst was then filtered, rinsed abundantly with distilled water, and dried at 80 °C in an oven.

### Fe coordination

C_2_N–Fe materials were prepared following the protocol reported by Fellinger and co-workers employing a wet impregnation method in methanol reflux.^36^ 60 mg of C_2_N-like material was placed in a 250 mL round bottom flask and dispersed in 75 mL MeOH. Subsequently, 75 mL of an FeCl_2_ solution in methanol (25 × 10^−3^ M, 98% Sigma Aldrich) were added, and the mixture was subjected to reflux for 24 h. Following the metalation reaction, the products were filtered, rinsed with methanol, and treated with 0.5 M H_2_SO_4_ (95–98% Sigma Aldrich) overnight to remove any Fe aggregated species. Finally, the obtained C_2_N *X*–Fe materials were rinsed thoroughly with distilled water and dried overnight at 80 °C.

### Electrochemical measurements

Electrochemical tests were performed without *iR* correction employing an AUTOLAB PGSTAT302N in N_2_ (≥99.99998% BIP® Plus, Air Products) and O_2_ (≥99.9998% UltraPure Plus, Air Products) saturated 0.1 M HClO_4_ (Suprapur®, Merck) electrolyte in a one compartment glass cell with three electrode configuration using Ag/AgCl_sat_ (3 M KCl) as reference electrode and a glassy carbon rod as a counter electrode. The 5 mm glassy carbon RDE working electrode (Metrohm) was first polished in a figure of eight to a mirror finish using a micropolish cloth and 0.05 μm alumina suspension (Buehlur). The catalysts prepared in this work were loaded on the freshly polished RDE by drop-casting 13 μL of an ink comprising 4 mg of the catalyst, 480 μL of 18.2 MΩ cm deionised water, 480 μL of isopropanol (≥99.5%, Honeywell™, Fisher Scientific) and 40 μL of 5 wt% Nafion® D-521 (5% w/w in water and 1-propanol, Alfa Aesar), leading to a catalyst loading of 0.26 mg cm^−2^. Drop-cast inks were dried at 700 rpm for at least 30 min while exposed to ambient air. Prior to measurement the reference electrode was calibrated in a separate cell filled with 0.1 M HClO_4_ (Suprapur) purged with 1 bar hydrogen 10 min prior to and during calibration. For calibration, a 3 mm Pt RDE tip (Metrohm) working electrode was rotated at 1600 rpm with the Ag/AgCl_sat_ and Pt rod (Metrohm) as reference and counter electrodes, respectively. 5 cyclic voltammograms were recorded at 10 mV s^−1^ between approximately −0.26 to −0.28 V *vs.* RHE and the average value of forward and backward scans at zero current was taken for conversion between Ag/AgCl_sat_ and RHE (example in Fig. S1†).^42^ Cyclic voltammograms under N_2_ or O_2_ were acquired after purging the electrolyte for at least 15 min and recorded at 50 mV s^−1^ when under 0 rpm for N_2_ saturated electrolyte only, and 10 mV s^−1^ when under 1600 rpm for both O_2_ and N_2_ saturated electrolyte. Capacitance was corrected for ORR measurements at 1600 rpm by subtracting N_2_ saturated cyclic voltammetry from the O_2_ saturated results. For the most active material (determined by the cathodic scan of cyclic voltammograms under O_2_ at 1600 rpm), electrochemical measurements under O_2_ at 1600 rpm were repeated 4 times. A commercial Fe-NC electrode (PMF-011904, Pajarito Powder) was used for comparison and was prepared in the same fashion but with an eventual loading of 0.2 mg cm^−2^. Meanwhile, a 40 wt% Pt/C catalyst (HiSPEC4000, Johnson Matthey) prepared with an overall Pt loading of ∼20 μg_pt_ cm^−1^, by drop-casting 10 μL of ink composed of 5 mg of 40 wt% Pt/C, 3.98 mL of deionised water, 1 mL of isopropanol and 20 μL of 5 wt% Nafion® D-521. Drop-cast Pt/C inks were dried at 700 rpm for at least 30 min while exposed to ambient air. Pt/C performance in this work is comparable to Garsany *et al.* stationary dried Pt/C inks exposed to air although not close to that of their inks dried at 700 rpm exposed to air (Fig. S2†).^43^ The kinetic current density (*j*_kin_) at 0.80 V_RHE_ was determined from the geometric disk current (*j*_d_) and geometric diffusion-limited current density (*j*_lim_) according to the Koutecký–Levich equation, eqn (1):1
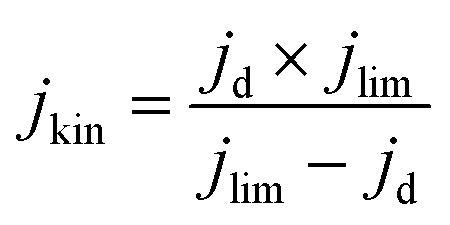


To calculate the number of electrons transferred, electrochemical measurements were carried out in a RRDE employing the same ink formulation, loading and procedures as stated previously for the RDE. The number of electrons transferred (*n*) was calculated from the disk and the ring current using eqn (2):^44^2
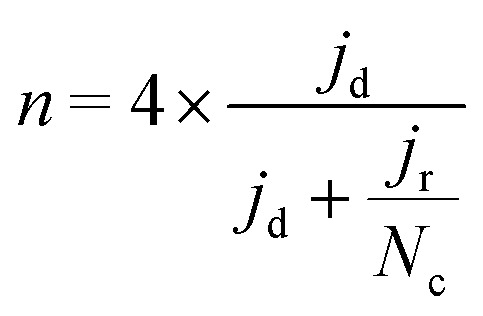
where *N*_c_ is the calculated theoretical collection efficiency (24.9%), *j*_r_ is the ring current. Additionally, the H_2_O_2_% was obtained from eqn (3):3
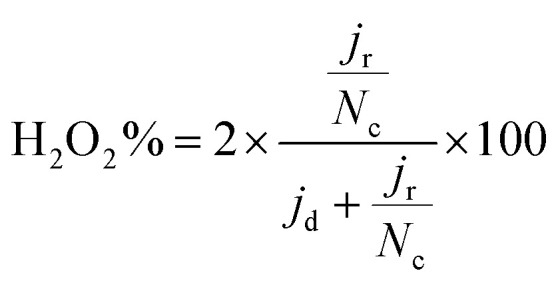


### Characterisation

XRD patterns were obtained with a powder X-ray diffractometer (PANanalytical X’PERT PRO). XPS analysis was conducted employing a Thermo Fisher K-Alpha XPS system, and the spectra analysed with the Avantage software. All spectra were calibrated relative to the carbon C 1s peak at 284.8 eV for correcting for charging effects. N_2_ sorption isotherms were conducted at −196 °C with data collected from pressure range 10^−5^ to 0.99 in Micromeritics 3Flex system with ∼10 mg of degassed sample (200 °C overnight). The BET surface area was deduced from the adsorption isotherm in the relative pressure range of <0.03 and the best region for linear fit as determined by the Rouquerol method^45^ on 3Flex software version 5.02. The pore size distribution was calculated from 0.36–50 nm by heterogeneous surface-2D-NLDFT method from the adsorption isotherm using SAIEUS software version 3.06. Raman spectra were collected using an inVia Renishaw confocal Raman microscope operated with an incident laser beam at 532 nm focused through a 50× objective (Leica). The laser intensity was minimised to 0.5 mW to avoid heating of the samples. For X-ray Absorption Spectroscopy (XAS) analysis, 14–18 mg samples (62–69 wt%) were well mixed with 6.4–10 mg (31–38 wt%) cellulose and pressed into 5 mm pellet with a thickness of 0.5–1 mm. XAS measurements were performed at beamline I20 of Diamond Light Source in Fluorescence mode. The data was normalised to the incident intensity and processed using the Athena software package.^46^ A standard Fe foil was used to calibrate the beam energy – a derivative (*E*_0_) value of 7112.0 eV corresponding to the first inflection point of the absorption K-edge. The optimised structures were calculated through FEFF 8 to obtain the paths. The measured EXAFS spectra were fit after subtracting pre-edge and post-edge background from the overall absorption and then normalising with respect to the edge step. Subsequently, the *χ*(*k*) data of 2.6 to 10.3 A^−1^ was used for the Fourier transformed data using a Hanning window (*d*_k_ = 1.0 Å^−1^) to separate the EXAFS contributions from different coordination shells around the absorbing Fe atom. To obtain the quantitative structural parameters around central atoms, least-squares curve parameter fitting was performed using the ARTEMIS software. Scanning Transmission Electron Microscopy (STEM) was carried out for the direct imaging of dual atoms in a JEOL ARM200F at 200 kV and the images analysed with the Gatan software. STEM samples were deposited onto the grids in dry condition to avoid solvent contamination. Briefly, 5 mg of powder and a STEM grid were placed in a glass vial, followed by gentle shaking to enable the catalyst to attach onto the grid electrostatically. The grid was then picked up and shaken with a tweezer to remove the excess powder on the surface and stored in dry conditions. Continuous wave electron paramagnetic resonance (EPR) was measured using an X-band CW ELEXSYS E500 EPR spectrometer (Bruker, Germany) with a cryogen-free variable temperature cryostat (Oxford Instruments, Oxfordshire, UK). Approximately 2 mg of sample was added to a 3 mm Wilmad quartz (CFQ) EPR tubes (Sigma Aldrich). Spectra were obtained at 5 K and 9.703 GHz with 20 mW incident microwave power, 100 kHz modulation frequency, 1 G modulation amplitude, 10 dB power attenuation and with magnetic field sweeping in 0.5 Gauss increments from 50 to 7000 Gauss. Inductively coupled plasma mass spectrometry (ICP-MS) was obtained using an Agilent 7900 spectrometer (Agilent Technologies). For ICP-MS, samples were first digested in 69% HNO_3_ (Certified AR, Eur.Ph., for analysis Fisher Chemical™, Fisher Scientific) by employing a MARS 6 microwave at 1500 W for 15 min at 215 °C. The resulting solutions were diluted to 2% HNO_3_ for measurement against calibration standards containing Fe concentrations of 0, 5, 50, 100, 200, 500 ppb.

## Results and discussion

**Scheme 1:**
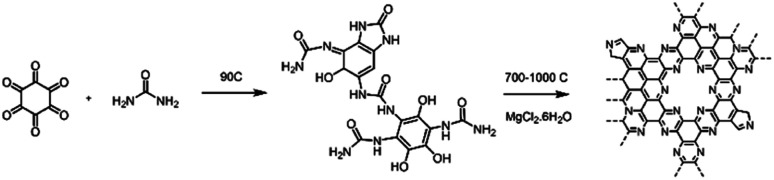
Synthetic pathway for the preparation of C_2_N-like materials.

C_2_N-Like materials were prepared by pyrolysis of a cross-linked complex comprising hexaketocyclohexane and urea in the presence of magnesium chloride at temperatures between 700 and 1000 °C (Scheme 1). X-ray diffraction (XRD) patterns confirm the amorphous nature of the materials, as in the diffraction pattern of C_2_N 700, a broad diffraction peak can be observed which corresponds to the interplanar (002) stacking peak of graphitic structures at 25.8° (Fig. S3a†). The annealing temperature is a very effective approach to control the chemical composition and structure of nitrogen-doped carbons.^47,48^ Raman measurements (Fig. S3b†) show a lower D/G band intensity ratio (*I*_D_/*I*_G_) for higher pyrolysis temperature, suggesting the formation of a defective graphene-like material with low nitrogen content at 1000 °C. This was further confirmed by evaluating the chemical composition of the C_2_N-like materials through X-ray photoelectron spectroscopy (XPS, Table S1†). At 700 °C the N 1s content is 20.2 at%, while at 1000 °C it drops to just 4.5 at% (17.2 at% for the material prepared at 800 °C and 8.5 at% for the material prepared at 900 °C). Despite the difference in nitrogen content, both C 1s and N 1s spectra show that the prepared carbons have a similar chemical state for the nitrogen species, displaying pyridinic, pyrrolic and graphitic species in the case of the materials prepared at 700–900 °C (Fig. S4 and S5†). Nevertheless, C_2_N 1000 shows a substantially lower amount of pyridinic moieties.

**Fig. 1:**
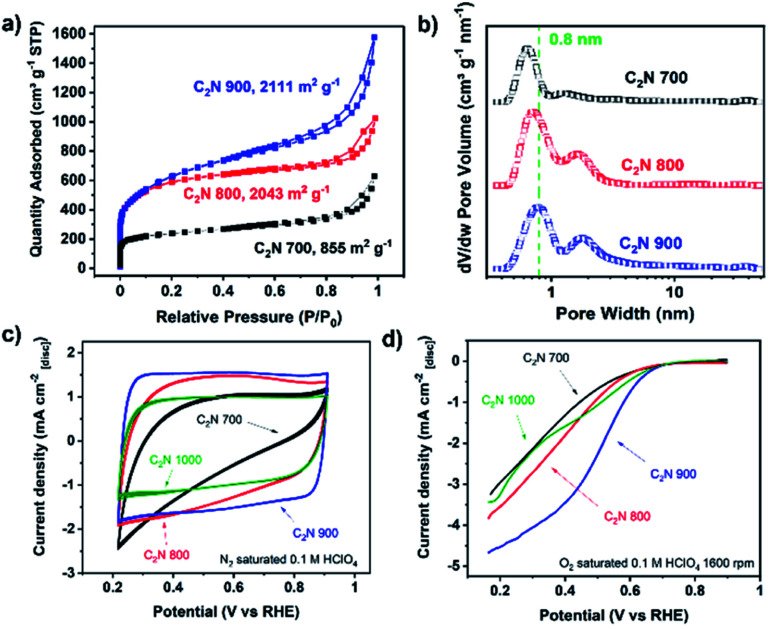
N_2_ physisorption isotherms (a), pore size distribution (b), cyclic voltammograms without rotation speed recorded at 50 mV s^−1^ (c), and capacitance-corrected cathodic scan of cyclic voltammograms at 1600 rpm of C_2_N-like materials recorded at 10 mV s^−1^ obtained by subtracting N_2_ saturated cyclic voltammetry at 1600 rpm to the O_2_ saturated (d). Electrochemical measurements conducted with no *iR* correction, 0.26 mg cm^−2^ catalyst loading and room temperature electrolyte.

Given that C_2_N 1000 has a low nitrogen content and lacks enough pyridinic moieties, we focused on the materials prepared at lower temperatures which showed more promising features for metals coordination. The utilisation of magnesium chloride hexahydrate as a porogen resulted in high specific surface area values arising from the microporosity when the pyrolysis temperature was 800 °C or higher,^49^ achieving 2043 and 2111 m^2^ g^−1^ in the case of C_2_N 800 and C_2_N 900, respectively. These values lead to higher accessibility of the nitrogen-rich pores for metals coordination in comparison with C_2_N 700 (Fig. 1a), whose specific surface area was 855 m^2^ g^−1^. Additionally, the pore size distribution confirms the existence of pores in the range of 8 Å (Fig. 1b), which agrees with the pore size of crystalline C_2_N-like networks reported by Mahmood *et al.* by means of scanning tunnelling microscopy;^38^ these porous features result in a very suitable binding site for single and dual atom catalysts with high diffusion barriers.^21,40^ The electrocatalytic activity towards the ORR of the metal-free C_2_N-like materials was evaluated with a RDE (Fig. 1c and d). Cyclic voltammograms show that a higher pyrolysis temperature, up to C_2_N 900, results in the enhancement of the capacitive current (Fig. 1c), suggesting a higher electrochemical surface area, whilst for C_2_N 1000 the capacitance is lower owing to the smaller nitrogen content.^50^ Amongst all the C_2_N-like materials, C_2_N 900 shows the lowest overpotential for the ORR (Fig. 1d). We note there are two competing factors that vary with temperature – materials synthesised at lower temperatures (C_2_N 700–800) show a very high nitrogen content that hinders their electrical conductivity,^48^ while C_2_N 1000 does not display enough nitrogen to create catalytic active sites.

**Scheme 2:**
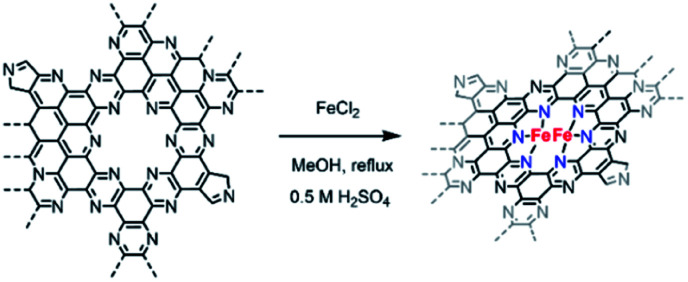
Schematic representation of the proposed metalation reaction in C_2_N-like materials.

Owing to the higher electrocatalytic performance of C_2_N 900, we selected this material for the metalation with FeCl_2_. Fe coordination on C_2_N 900 was carried out as previously described by Fellinger and co-workers for single atom catalysts (Scheme 2).^36,51^ The reaction of C_2_N 900 with FeCl_2_ in methanol under reflux led to the formation of well-defined Fe atomic species. XPS and ICP results showed that Fe loadings up to 1.7 wt% could be obtained in the C_2_N materials employing this technique (Fig. S6 and Table S2†). The chemical states of N 1s in C_2_N 900 slightly change as a result of the pyridinic N–Fe coordination, which leads to a new contribution at 398.9 eV with 18.6 at% (Fig. S7†). XRD patterns shows no evidence of any large Fe-based nanoparticles after the metalation reaction (Fig. S8†). We note, that despite the pyrrolic moieties present in the surface, the pyridinic nitrogens (in the form of pyrazine) are the main coordination site for Fe atoms according to XPS; additionally, a pyrrolic coordination would lead to low-coordinated Fe species in the grain boundary, which are prone to aggregation and less resistant to acid washing.

**Fig. 2:**
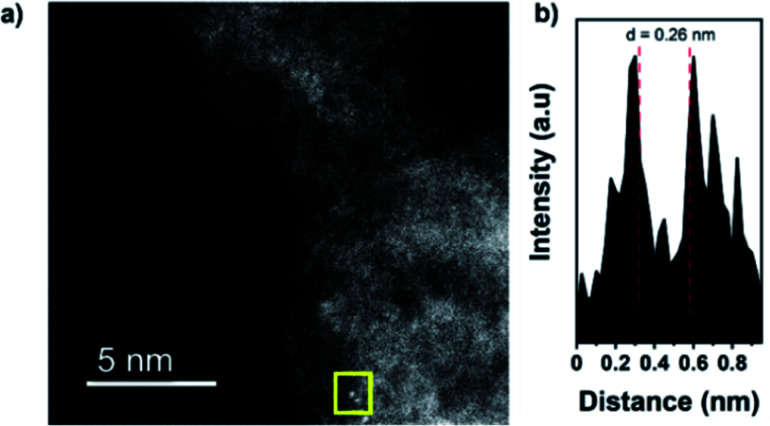
HAADF-STEM image (before electrochemical measurements) of C_2_N 900@Fe (a), and intensity profile of the atomic site highlighted (b). STEM images of clusters can be found at Fig. S12.†

High angle annular dark-field scanning transmission electron microscopy (HAADF-STEM) images confirm the presence of isolated dimers in C_2_N 900@Fe with interatomic distances of 0.26 and 0.27 nm (distances calculated from the intensity profile of different diatomic sites employing Gatan software, Fig. 2a and b and S9–S12†)^52^ but showing as well the presence of low-nuclearity clusters composed of Fe of up to 2–3 nm (Fig. S13†). To investigate further the atomic structure of the obtained samples, X-ray absorption spectroscopy (XAS) was employed to obtain insights on the coordination environment of the Fe atoms.

**Fig. 3:**
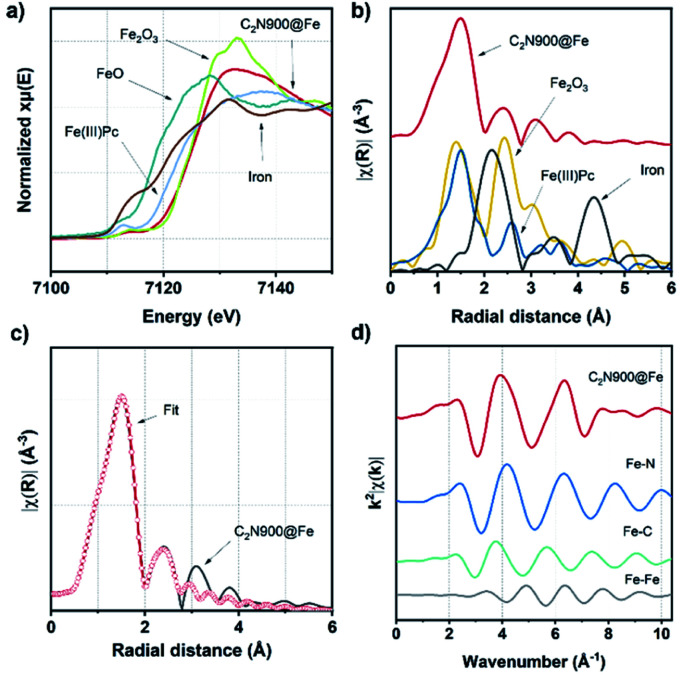
XANES spectra (before electrochemical measurements) of C_2_N900@Fe, Fe(iii)Pc, Fe_2_O_3_, FeO and Fe foil (a). FT of Fe K-edge EXAFS spectra of C_2_N900@Fe, FePc, Fe_2_O_3_, FeO and Fe foil (b). The magnitude of EXAFS FT *k*_2_-weight Fe K-edge spectra and fitting curve of C_2_N900@Fe (c) and *q* space of C_2_N900@Fe and Fe–N, Fe–C, and Fe–Fe paths (d).

By comparison of the X-ray absorption near edge structure (XANES) to measured standards (Fig. 3a and S14a†) we observed that the white line of both samples show none of the distinctive features of the Fe_2_O_3_, suggesting the absence of Fe_2_O_3_ particles at least within the sensitivity of this experiment.

Additionally, extended X-ray absorption fine structure (EXAFS, Fig. 3b and S14b†) Fourier Transforms (FT) of C_2_N 800@Fe and C_2_N900@Fe display strong single peaks centred around 1.5 Å, which differ significantly from those of metallic Fe foil, Fe_2_O_3_ and are similar to the iron phthalocyanine reference (FePc). This observation, along with the nitrogen-rich character of the materials (as observed with XPS), suggests the formation of well-defined Fe–N_4_ coordinated sites (but does not preclude coordination to other species such as O or C).^53^ Wavelet transform (WT) EXAFS was employed to elucidate the radial distance resolution and *k* space resolution which can provide insights on the catalyst active site structure.^54^ As shown in Fig. S15,† WT of C_2_N 800@Fe and C_2_N900@Fe show one main intensity maximum at ∼4.1 Å^−1^, which is very close to that in the reference FePc (∼4.2 Å^−1^). Furthermore, C_2_N 800@Fe and C_2_N900@Fe show a weaker peak at ∼4.4 Å^−1^ which potentially could be assigned to Fe–Fe bond originating from the dual atom configuration, suggesting a mixture between single and dual site catalysts.

To further study the atomic configuration of the Fe sites, FT EXAFS fittings were performed for C_2_N 800@Fe and C_2_N900@Fe (Fig. 3c, S14c–f and S16†). The best-fit values (Table S3†) for the EXAFS modelling of C_2_N 900@Fe and C_2_N 800@Fe requests three paths in the fitting (Fig. 3d and S14d,† fitting details could be found in Table S3†), which provide an average coordination number of 1.34 for the Fe–Fe species with a bond distance of 2.55 Å, consistent with the STEM data in Fig. 2.

This further supports the formation of a dual atom site along with aggregated species, as if Fe only existed in the dual site the Fe–Fe coordination number would be one, whilst in aggregated Fe it would be 6–8.^55^ Consequently, our data suggest that in addition to the dual atoms there are some clustered species, as shown in Fig. S13.† For Fe–N bond, a coordination number of 3.58 was found with a bond distance of 2.02 Å. This value is consistent with the notion that C_2_N 900@Fe is comprised of dual atom catalysts with Fe–N coordination number three and single atomic species with coordination number four.^56,57^

**Scheme 3:**
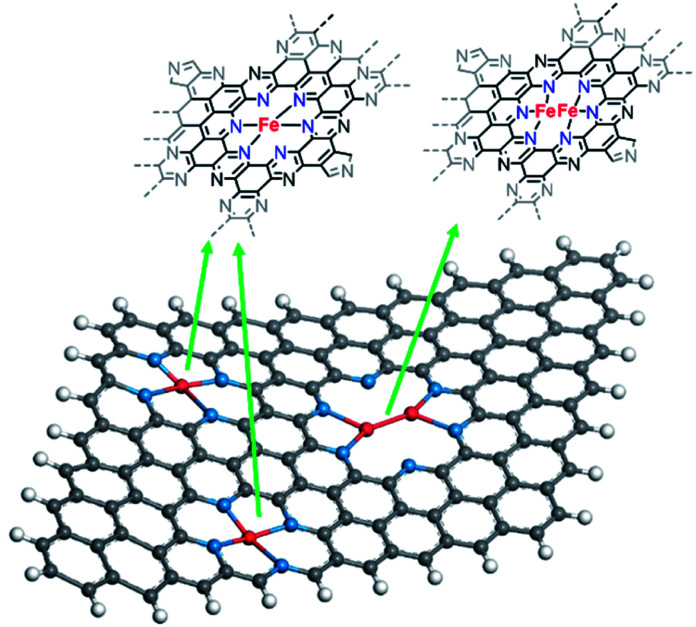
Proposed structures for the mixture of dual atom and single atom within the C_2_N 900 sample based on EXAFS analysis (spheres with different colour represent: red – iron, blue – nitrogen, grey – carbon, white – hydrogen).

From XAS analysis we can therefore hypothesize that the prepared catalysts consist of a mixture of single metal sites, dual metal sites (Scheme 3) and some larger aggregates containing Fe. Finally, Fe–C bond with 2.25 coordination number and Fe–C bond distance of 2.26 Å was observed. Meanwhile in the case of C_2_N800@Fe, Fe–N and Fe–C remained similar and the main difference was found in the average coordination numbers for the Fe–Fe bond; 0.42 at 2.54 Å. The presence of Fe–Fe with near one coordination number and bond distance of 2.54 Å suggests the formation of a dual metal atom catalyst. Additionally, the Fe–N coordination is lower than 4, which may be due to a mixture between single and dual active atoms with different Fe–N coordination number (Scheme 3). Comparing C_2_N 800@Fe and C_2_N900@Fe, we observed higher Fe–Fe coordination in the latter, suggesting a higher amount of dual metal atom sites in C_2_N900@Fe. Therefore, based on the HAAD-STEM and XAS results, we propose that iron exists as a mixture of single and dual atom sites (Scheme 3) and potentially some aggregated iron in the form of Fe_3_C.

To further study the local environment of Fe centres, cryo electron paramagnetic resonance (EPR) was employed to probe the ligand geometry of these sites (Fig. S17†). The dominant signal of C_2_N 900@Fe at *g* ∼ 4.3 is attributed to high spin Fe^3+^ with rhombic zero field splitting,^58^ meanwhile a small signal at *g* ∼ 8.8 for C_2_N900@Fe is designated to Fe^3+^ with quasi-octahedral coordination.^59^

The sharp *g* ∼ 2 signal seen in both C_2_N 900@Fe and C_2_N 900 is ascribed to organic radicals in the carbon matrix,^58^ since the latter does not possess Fe^3+^ sites. Interestingly, the *g* ∼ 2 signal is reduced from C_2_N 900 to C_2_N 900@Fe suggesting the metalation reaction or acid washing process reduces organic radical species.

**Fig. 4:**
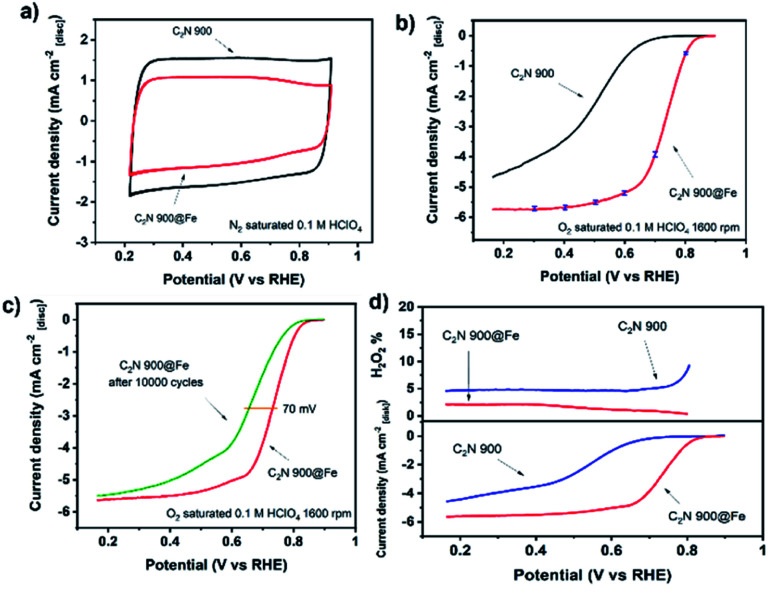
Cyclic voltammogram of C_2_N 900 before and after Fe metalation recorded at 50 mV s^−1^ (a). Capacitance-corrected cathodic scan of the cyclic voltammogram in 0.1 M HClO_4_ with a rotation speed of 1600 rpm recorded at 10 mV s^−1^ obtained by subtracting N_2_ saturated cyclic voltammetry at 1600 rpm from the O_2_ saturated result. For C_2_N 900@Fe, three independent measurements were carried out with average value plotted. Error bars correspond to largest deviating value from average at the given potential. (b). Cathodic scans of the cyclic voltammograms at 1600 rpm before and after 10 000 cycles between 0.6 and 0.9 V recorded at 100 mV s^−1^ under N_2_ saturation (c). H_2_O_2_ production from cathodic scan RRDE measurement for C_2_N 900 and C_2_N 900@Fe at 1600 rpm, 10 mV s^−1^, capacitance correction and under O_2_ saturation (d). All measurements conducted with no IR correction, 0.26 mg cm^−2^ catalyst loading and room temperature electrolyte.

The electrochemical performance of C_2_N 800@Fe and C_2_N 900@Fe for the oxygen reduction reaction was evaluated in N_2_ and O_2_ saturated 0.1 M HClO_4_. The limiting current reached values comparable to that of a planar Pt electrode.^56,60^ Despite the substantial improvement in the electrochemical performance of C_2_N 800 after metal coordination (Fig. S18†), the lower electrical conductivity of the metal-free support (owing to the 17.1 at% of nitrogen within the structure) hinders the overall activity resulting in a kinetic current and mass activity of *J*_kin_ = −0.05 mA cm^−2^ and 0.05 A g^−1^ respectively, and half-wave potential of *E*_1/2_ = 0.64 V. After loading Fe in C_2_N 900, the capacitance slightly decreased, putatively due to the blocking of N-rich pores by Fe atoms (Fig. 4a). At 1600 rpm, C_2_N 900@Fe showed a high activity, displaying a kinetic current density at 0.8 V of *J*_kin_ = −0.63 ± 0.08 mA cm^−2^, mass activity of 2.4 ± 0.3 A g_carbon_^−1^ and a half-wave potential of *E*_1/2_ = 0.73 V which entails a substantial improvement *versus* that of a commercial Fe-NC catalyst (mass activity 1 A g^−1^, *E*_1/2_ = 0.65 V, Fig. S19†) and the metal-free counterpart (Fig. 4b). There was a negligible production of hydrogen peroxide (electron transfer number >3.95, Fig. 4d, S20a and b†). This performance lies within the best performing single and dual atom catalysts published in literature (Fig. S21 and Table S4†).

The stability of the catalyst was assessed by recording the cyclic voltammograms after 10 000 cycles in N_2_ saturated electrolyte between 0.6 and 0.9 V *vs.* RHE (Fig. 4c). C_2_N 900@Fe showed moderate stability with a decrease of 70 mV in half-wave potential. While the activity is amongst the highest in the literature, we cannot definitively ascertain, at present, whether the dual metal atom catalysts atom sites are significantly more active than single atom counterparts. Further work is underway to quantify the number of dual sites that are present and to determine their structure under reaction conditions.

## Conclusions

In summary, we have synthesised a dual metal atom catalyst (combined with some single atom catalysts and Fe-containing nanoparticles), which we supported in a C_2_N-like material. The material was prepared by a two-step procedure comprising the synthesis of a tailored N-doped carbon support followed by controlled metal coordination. The catalyst exhibits a high mass activity, comparable to the best in the literature and significantly better than a commercial non-precious metal catalyst. The pyrolysis temperature allows fine tuning of the structure and composition of the metal-free support; and Fe insertion significantly improves the activity. Additionally, the high nitrogen content in the vicinity of the pore allows the formation of dual metal atom sites as well as isolated Fe–N_4_ and small Fe clusters. Future work will focus on determining the intrinsic activity of the active site, as well as identifying the structure formed *in situ*. Our method provides a tailorable route towards the synthesis of dual atom catalyst for multiple electrochemical energy conversion applications.

## Author contributions

J. B. and A. P. contributed equally to this work. J. B. co-conceived the work, designed the synthesis of the materials, obtained XRD and XPS data, performed ORR measurements and wrote the initial draft. A. P. performed ORR measurements, N_2_ adsorption, ICP, and EPR measurements. J. F. assisted with RRDE measurements and obtained XAS data and fitting. S. C. S. assisted with XAS analysis and ORR measurements. M. W. assisted with ORR measurements. A. L. recorded and analysed STEM images. H. Y. obtained Raman data. H. L. assisted with STEM and XAS sample preparation. All authors contributed to interpretation of the data. M. P. R. provided supervision, revised, and edited the final manuscript. M.-M. T. co-conceived the work, co-supervised the work, revised, and edited the final manuscript. I. E. L. S. co-conceived the work, supervised the electrochemistry measurements and the characterisation, and revised and edited the final manuscript.

## Conflicts of interest

There are no conflicts to declare.

## Supplementary Material

TA-010-D1TA09560A-s001
