# Supplementary material for: Metal coordination in C2N-like materials towards dual atom catalysts for oxygen reduction
Source: J Mater Chem A Mater. 2022 Feb 11;10(11):6023–30. doi: 10.1039/d1ta09560a (PMC8922559; doi:10.1039/d1ta09560a)
Supplement: TA-010-D1TA09560A-s001 [file TA-010-D1TA09560A-s001.pdf]

Electronic Supplementary Material (ESI) for Journal of Materials Chemistry A.
This journal is © The Royal Society of Chemistry 2022

Supporting Information for:

Metal Coordination in C_2N-like Materials Towards Dual Atom Catalysts for Oxygen Reduction

Jesús Barrio,[a] Angus Pedersen,[a] Jingyu Feng,[b] Saurav Ch. Sarma,[b] Mengnan Wang,[a] Alain Li,[b] Hossein Yadegari,[a] Hui Luo,[b] Mary P. Ryan,[a] Maria-Magdalena Titirici*[b,c] and Ifan. E. L. Stephens*[a]

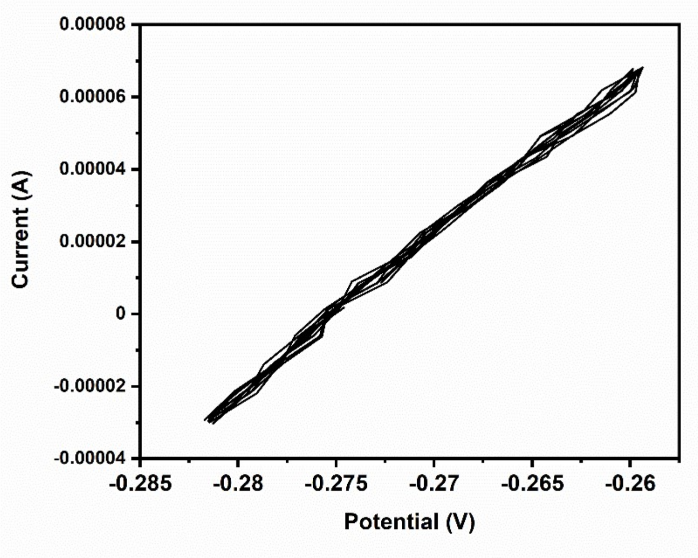

Figure S1. Example calibration of Ag/$AgCl_{sat}$ electrode in 0.1 M $HClO_4$. Cyclic voltammetry (10 mV s^{-1}) under 1 bar hydrogen purging with a 3 mm Pt RDE tip working electrode at 1600 rpm with Ag/AgCl and Pt rod as reference and counter electrode, respectively.

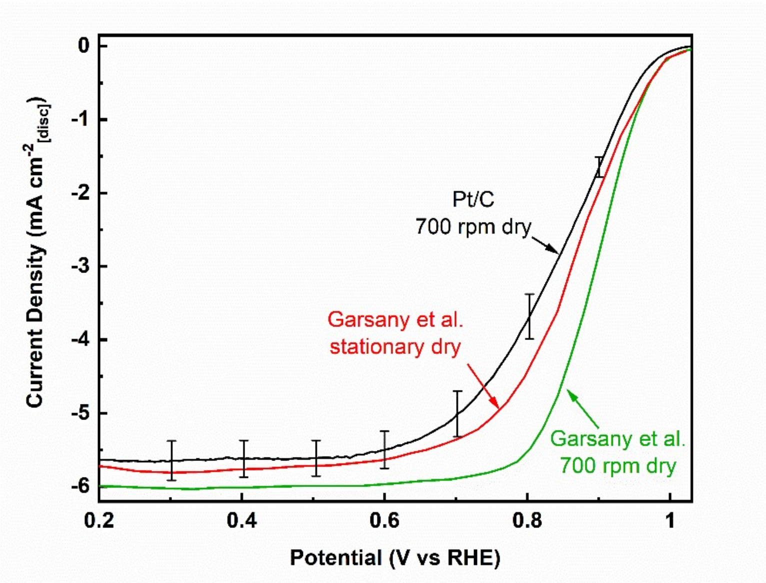

Figure S2. Comparison of the average ORR polarization curves from Garsany et al.[1] (30°C, 1600 rpm, 20 mV s^{-1}, anodic sweep, background correction, no iR correction) to average polarization curve (from five independent films) in this work (room temperature, 1600 rpm, 10 mV s^{-1}, anodic sweep, background correction, no iR correction). Error bars calculated from standard deviation of the five independent films. In this work and Garsany et al.,[1] 40 wt% Pt/C (Johnson Matthey HiSpec4000) with final Pt loading of ~20 μg_{Pt} cm^{-2} was measured in O_2-purged 0.1 M $HClO_4$ electrolyte.

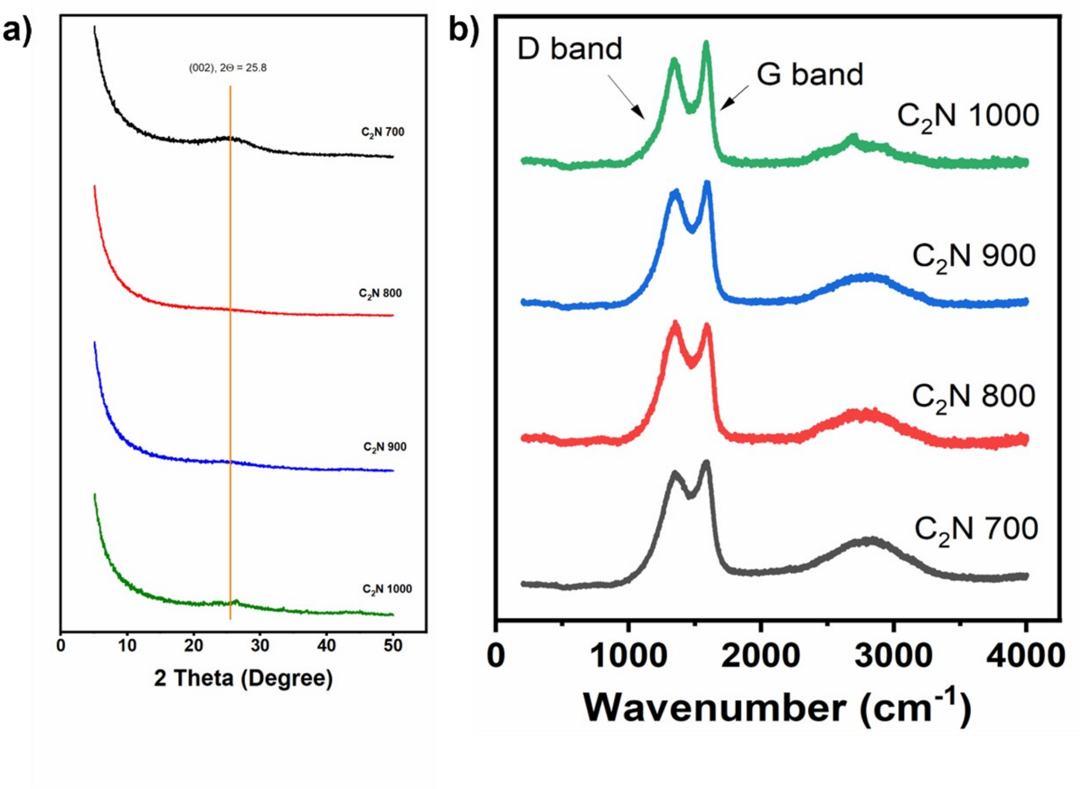

Figure S3. XRD patterns (a) and Raman spectra (b) of C_2N-like materials.

Table S1. Chemical composition of C_2N-like materials obtained by XPS.

Element	Atomic % 700	Atomic % 800	Atomic % 900	Atomic % 1000
C1s	70.84	78.88	88.0	92.0
N1s	**20.19**	**17.18**	**8.46**	**4.48**
O1s	8.97	3.94	3.54	3.20

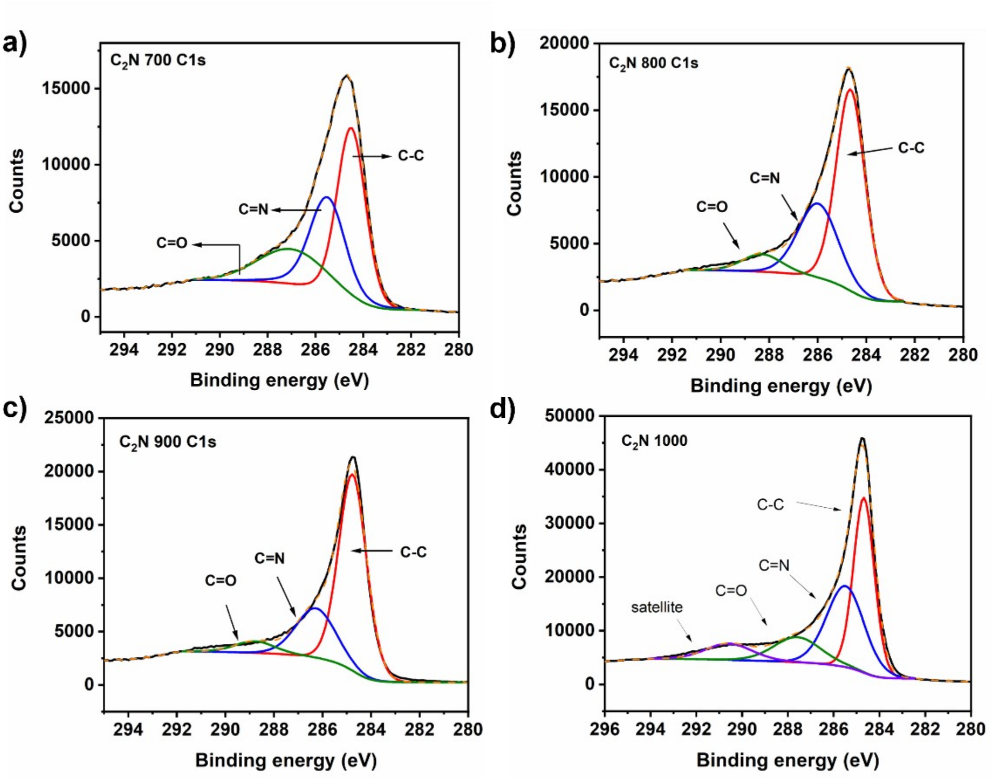

Figure S4. C1s XPS spectrum for the prepared C_2N-like materials at different temperatures.

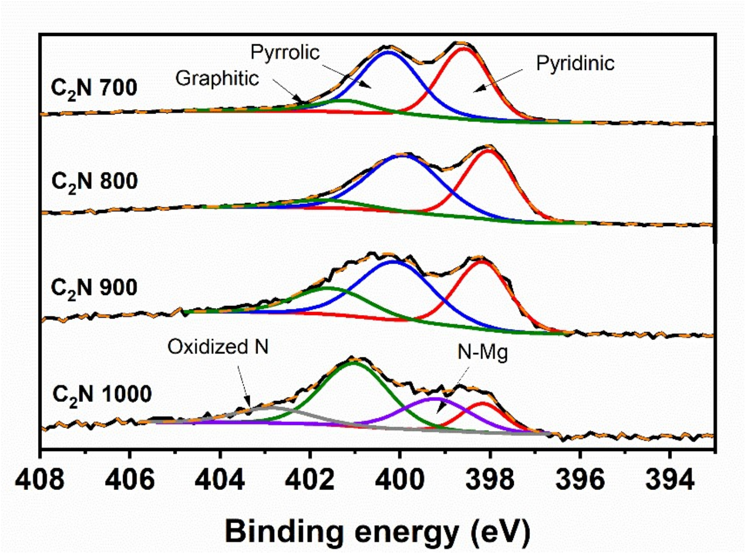

Figure S5. N1s XPS spectrum for the prepared C_2N-like materials at different temperatures.

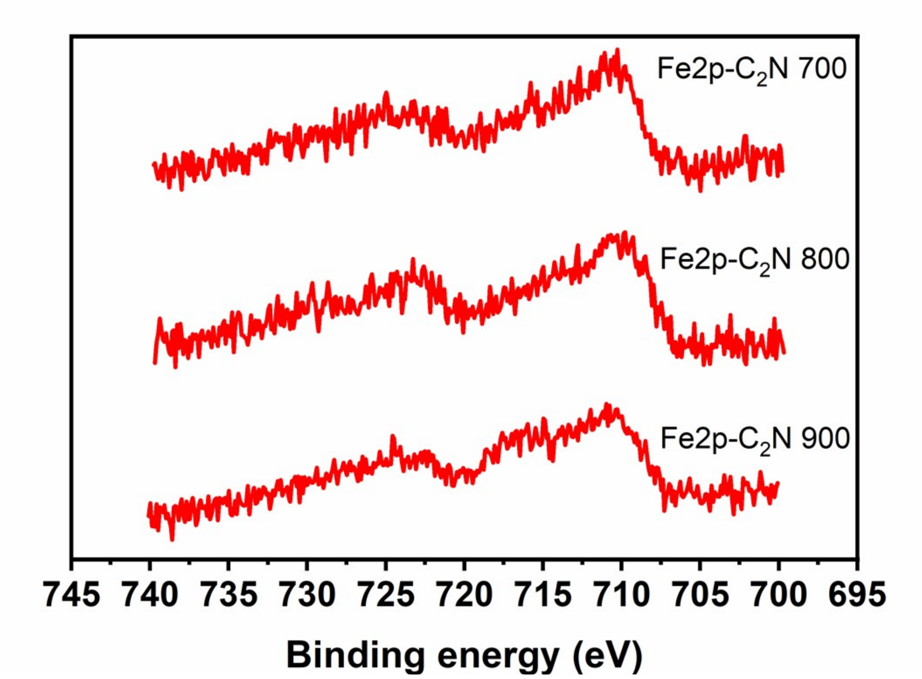

Figure S6. XPS Fe 2p spectrum for the C_2N-Fe materials after metalation.

Table S2. Fe content of C_2N-like materials after metalation obtained by XPS and ICP.

Material	Fe wt % (XPS)	Fe wt % (ICP)
C_2N 700@Fe	1.7	1.65
C_2N 800@Fe	2.0	1.3 ± 0.1
C_2N 900@Fe	1.3	0.7 ± 0.03

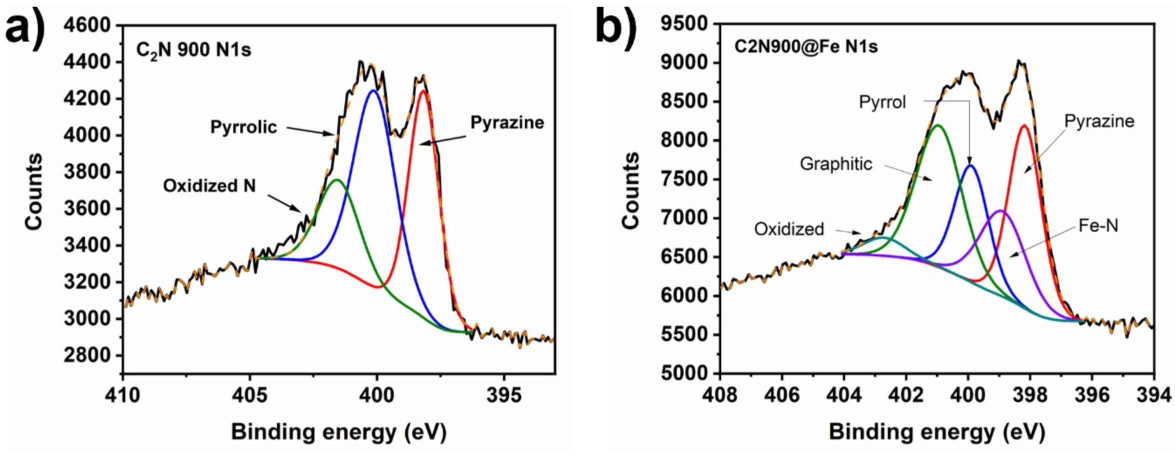

Figure S7. N1s XPS spectra for C_2N 900 and C_2N900@Fe.

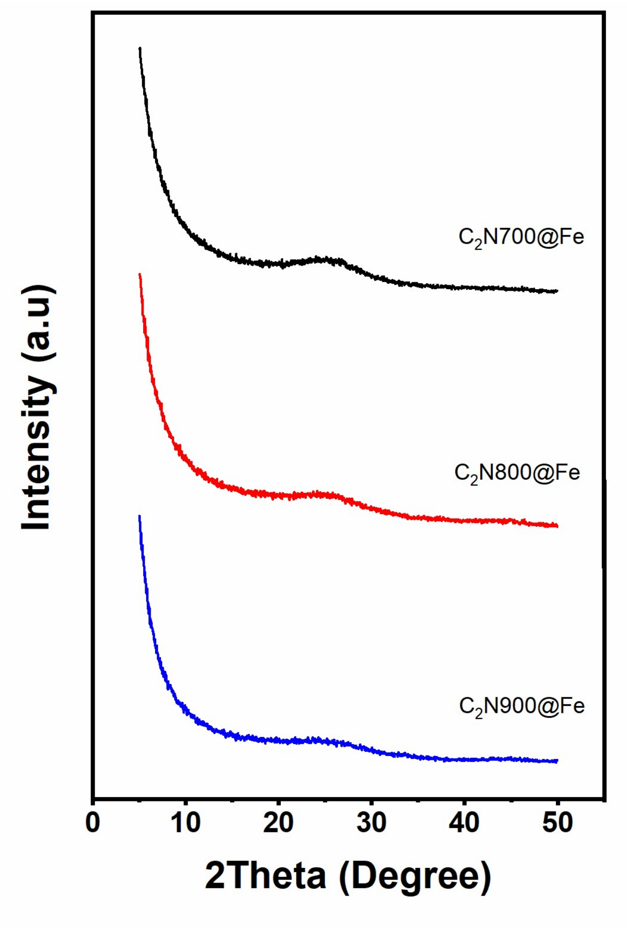

Figure S8. XRD patterns of C_2N-like materials after Fe metalation.

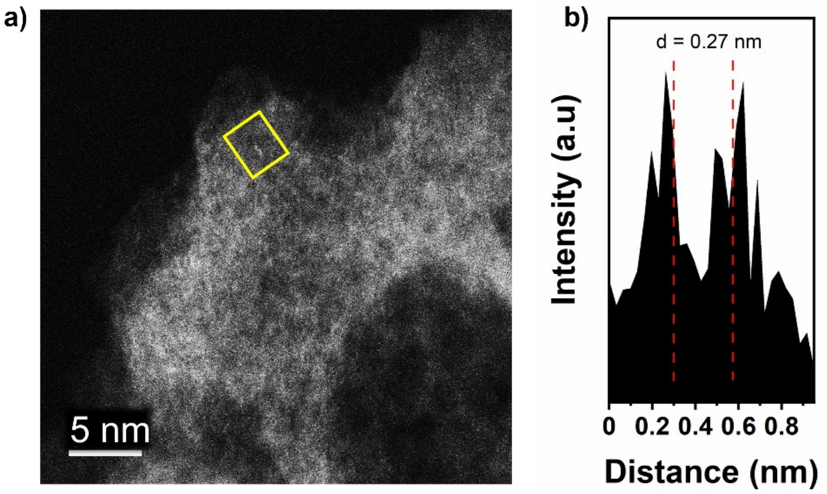

Figure S9. HAADF-STEM image of C_2N 900@Fe (a) and intensity profile of the atomic site highlighted (b).

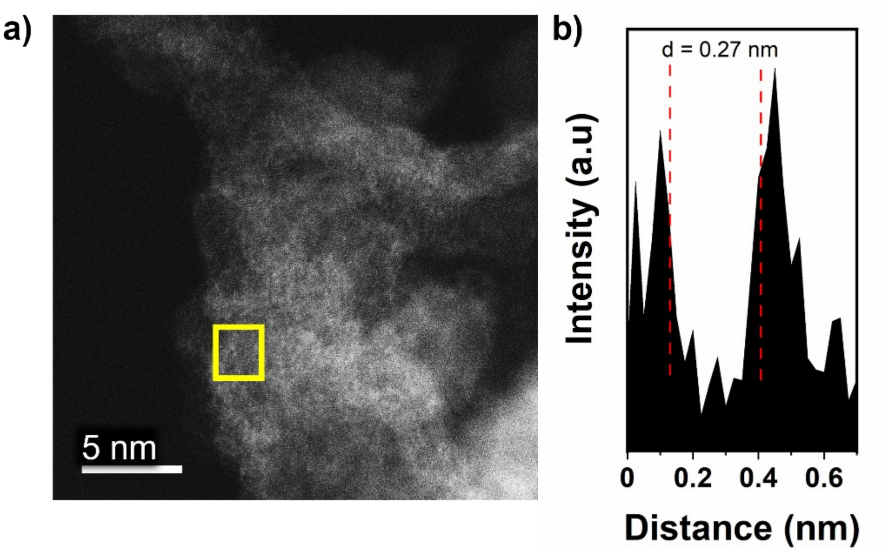

Figure S10. HAADF-STEM image of C_2N 900@Fe (a) and intensity profile of the atomic site highlighted (b).

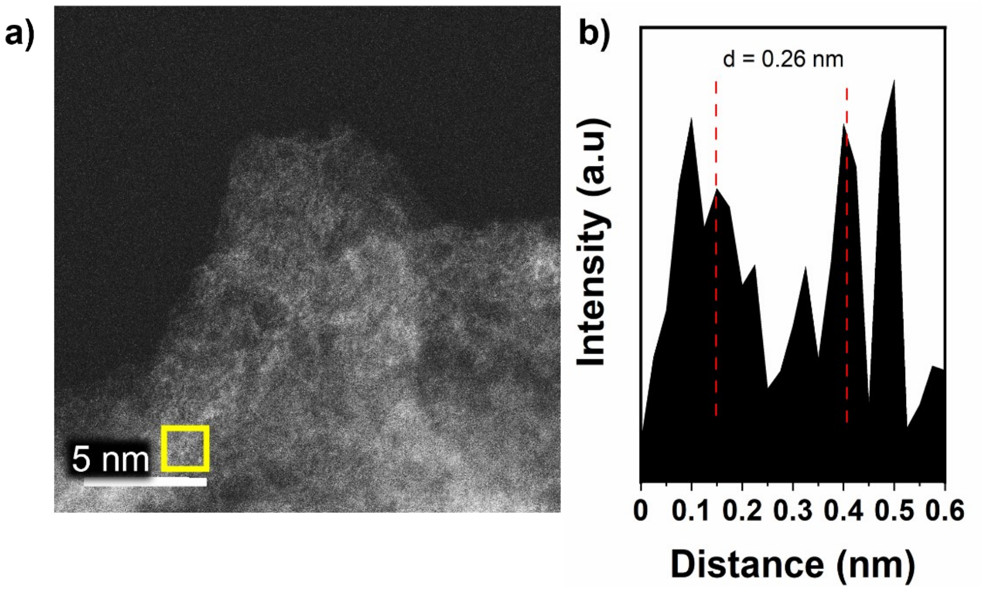

Figure S11. HAADF-STEM image of C_2N 900@Fe (a) and intensity profile of the atomic site highlighted (b).

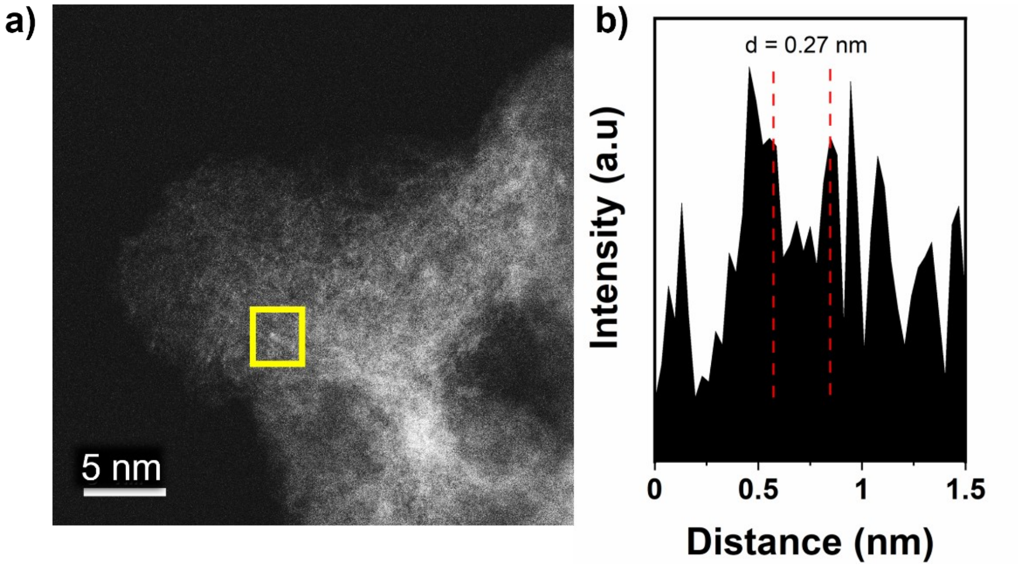

Figure S12. HAADF-STEM image of C_2N 900@Fe (a) and intensity profile of the atomic site highlighted (b).

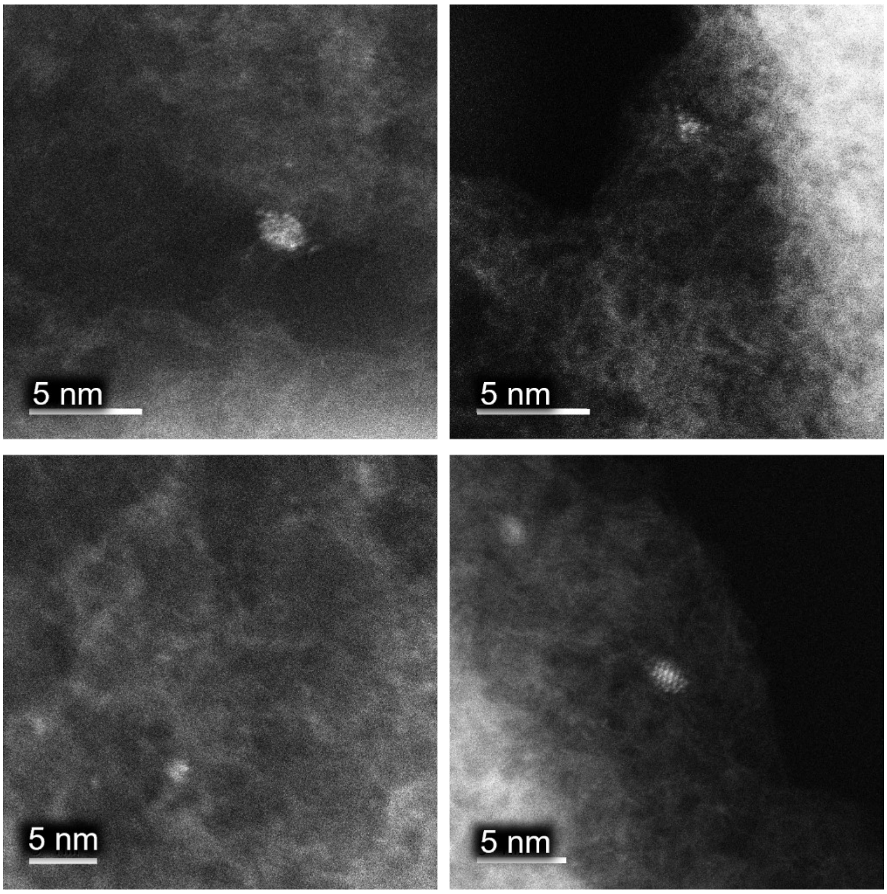

Figure S13. HAADF-STEM images of C_2N 900@Fe showing low nuclearity Fe clusters.

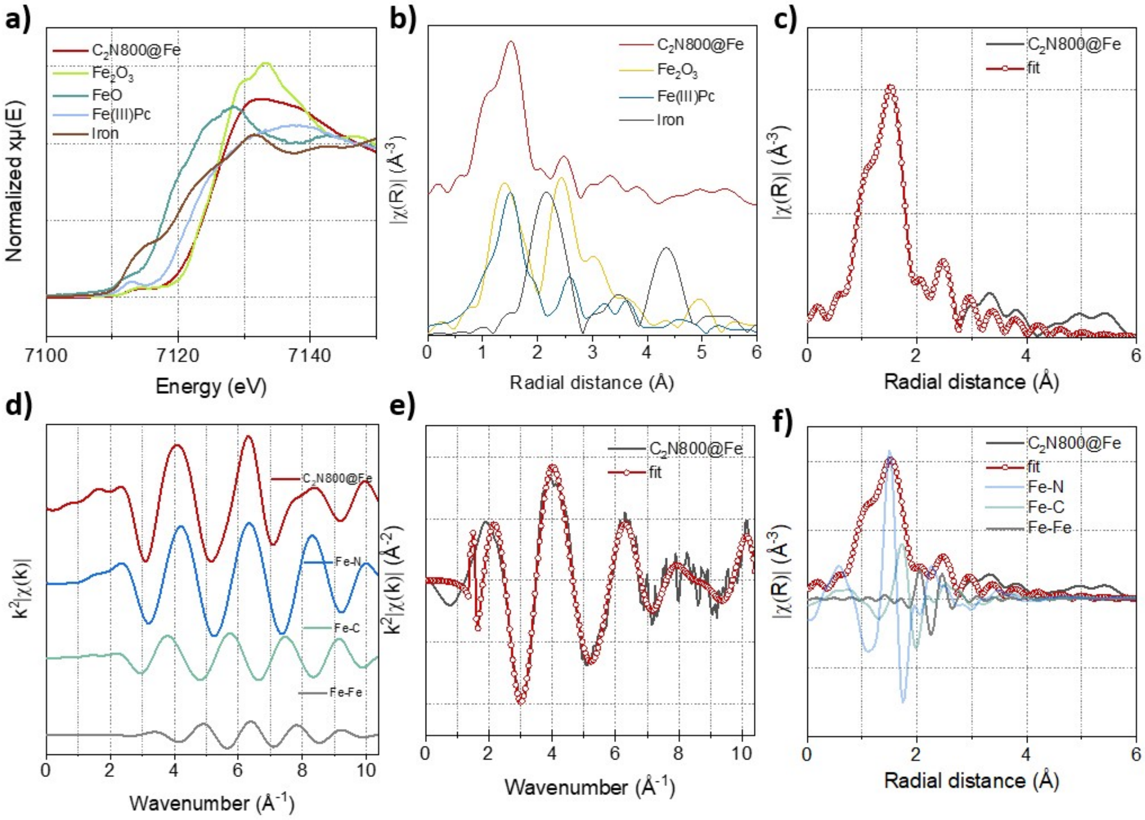

Figure S14. (a) XANES spectra of $C_2N800@Fe$, Fe(III)Pc, Fe_2O_3, FeO and Fe foil. (b) Fourier transform of Fe K-edge EXAFS spectra of $C_2N800@Fe$, Fe(III)Pc, Fe_2O_3, FeO and Fe foil. (c) The magnitude of EXAFS FT k2-weight Fe K-edge spectra and fitting curve of $C_2N800@Fe$, (d) EXAFS spectra of $C_2N800@Fe$ and Fe-N, Fe-C, and Fe-Fe paths, (e) k-space fitting curve of $C_2N800@Fe$, (f) The magnitude of EXAFS FT k2-weight Fe K-edge spectra and fitting contributions from Fe-N, Fe-C, and Fe-Fe paths.

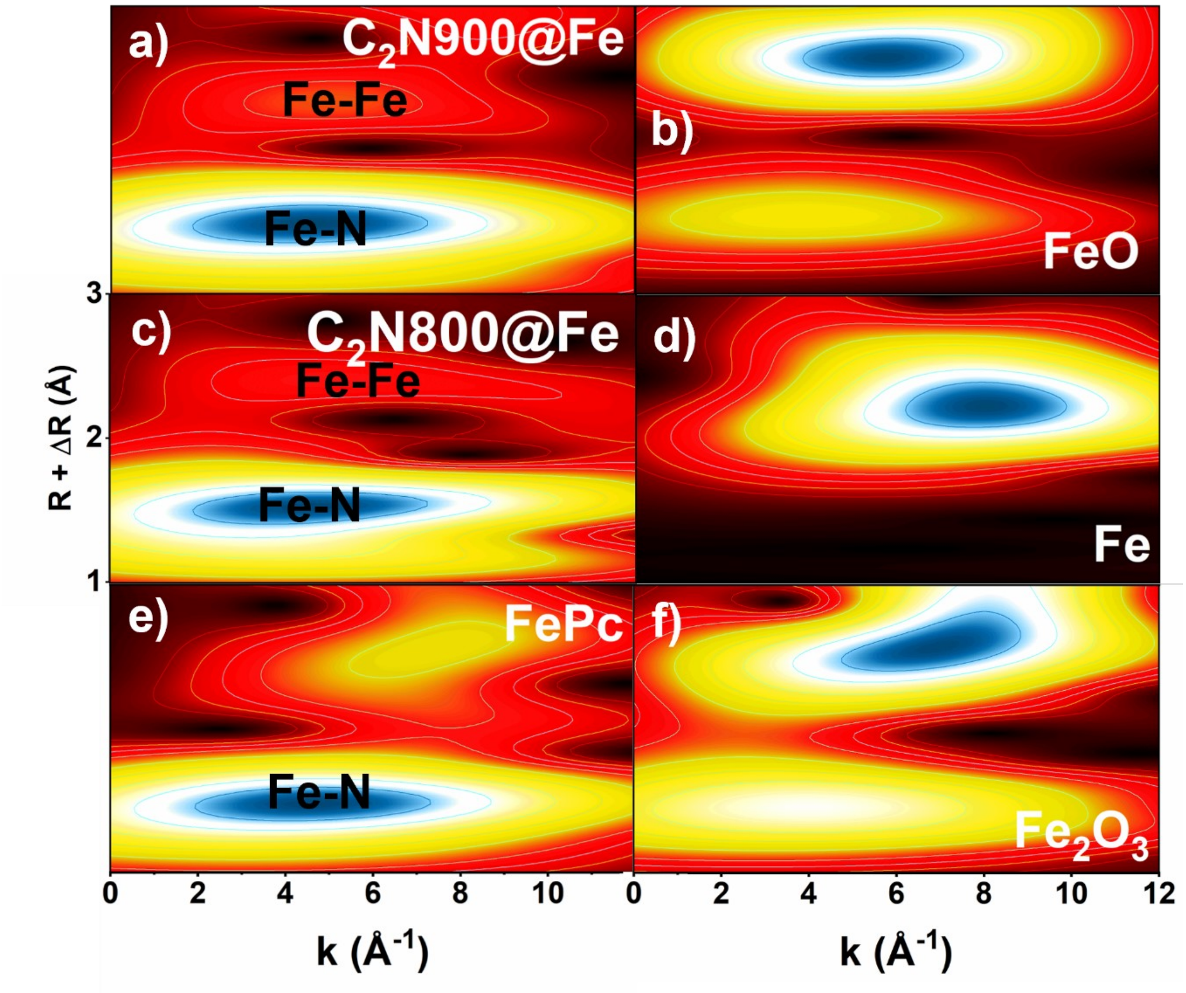

Figure S15. Wavelet transform of the k2 weighted EXAFS data of $C_2N800@Fe$, $C_2N900@Fe$, Fe(III)Pc, Fe_2O_3, FeO and Fe foil.

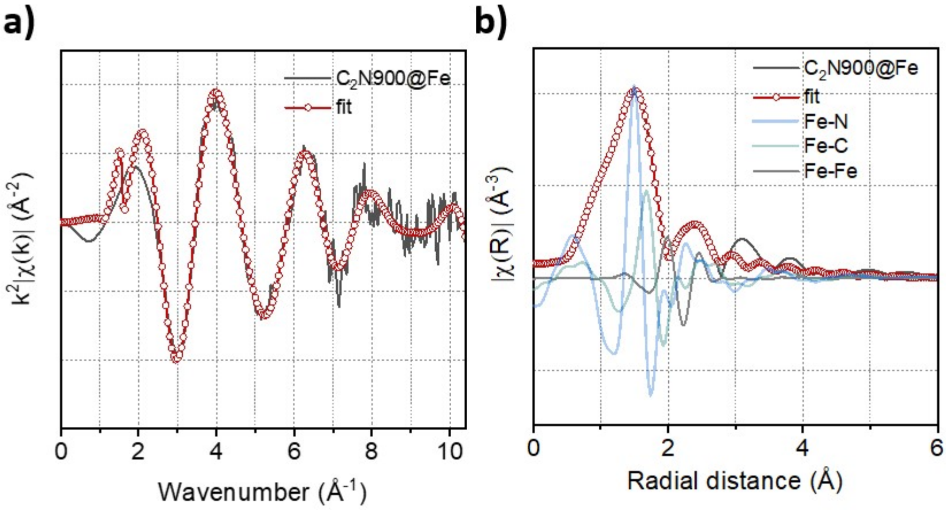

Figure S16. (a) k-space fitting curve of $C_2N900@Fe$, (b) The magnitude of EXAFS FT k2-weight Fe K-edge spectra and fitting contributions from Fe-N, Fe-C, and Fe-Fe paths.

Table S3. XAS fitting parameters for $C_2N800@Fe$ and $C_2N900@Fe$. S02 is obtained by fitting iron foil

Samples	Scattering pair	CN	R (Å)	ΔE_0 (eV)	σ^2 (10^{-3}Å^2)	$S0^2$	R factor
40590-C2N-Fe800	Fe-N	3.71	2.01	7.01 +/- 2.10	3.50	0.91	1.12
		+/- 0.38	+/- 0.02				
	Fe-C	2.00	2.23		3.00		
		+/- 0.42	+/- 0.02				
	Fe-Fe	0.42	2.54		13.00		
		0.15	+/- 0.02				
40590-C2N-Fe900	Fe-N	3.58	2.02	4.72+/-2.95	3.50	0.91	2.04%
		+/- 0.64	+/- 0.03				
	Fe-C	2.25	2.26		3.00		
		+/- 0.74	+/- 0.03				
	Fe-Fe	1.34	2.55		13.00		
		+/- 0.54	+/- 0.04				

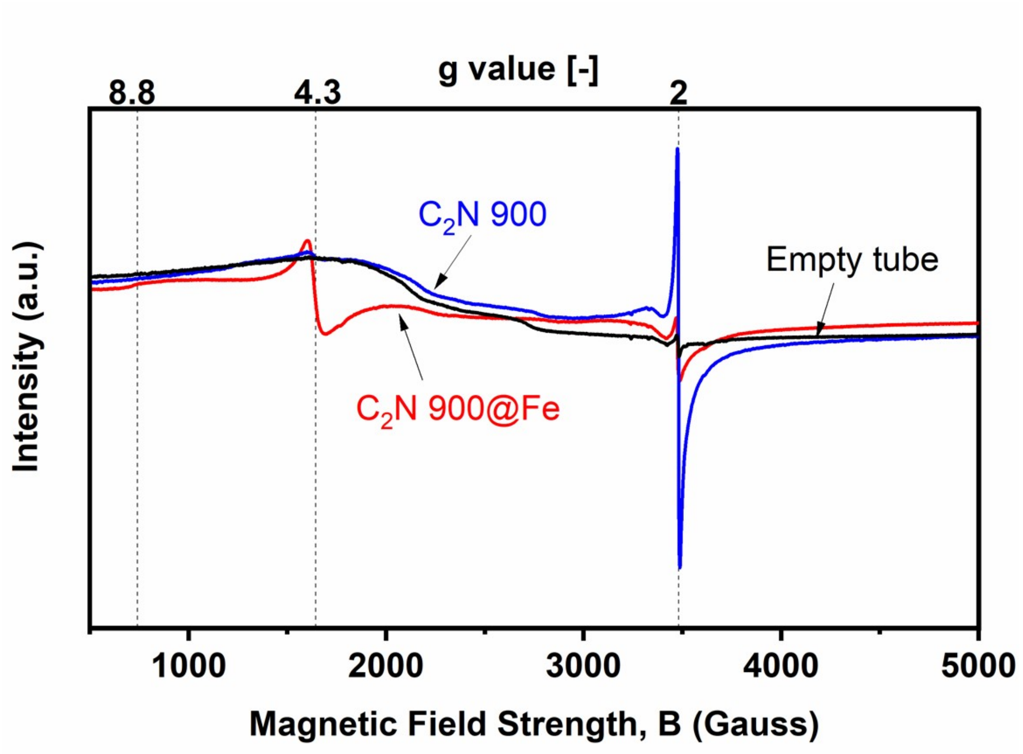

Figure S17. X-band cryo EPR (5 K) of C_2N 900, C_2N 900@Fe and empty quartz tube.

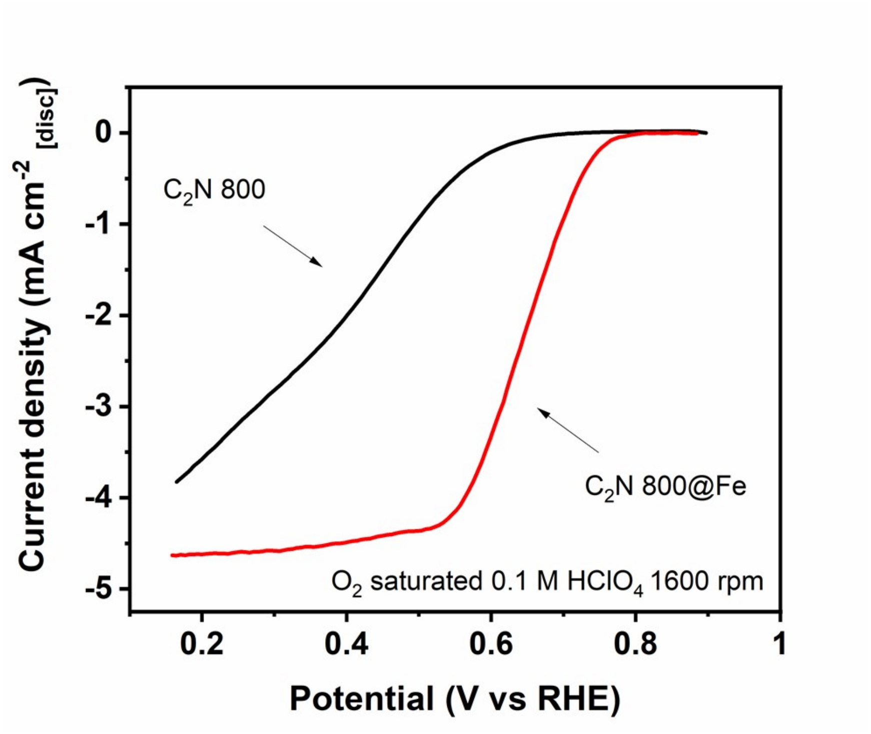

Figure S18. Capacitance corrected cathodic scan of cyclic voltammogram with a rotation speed of 1600 rpm of C_2N 800 before and after Fe metalation recorded at 10 mV s^{-1}.

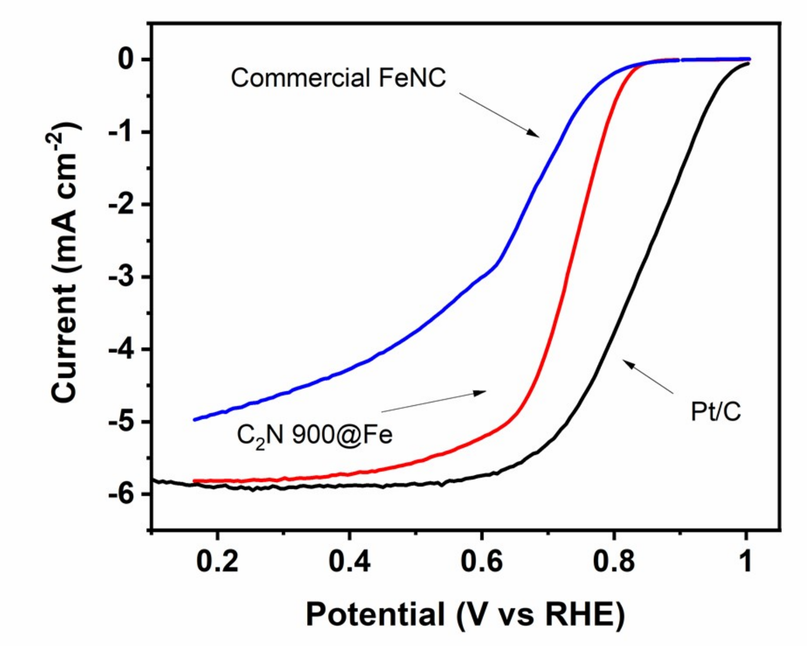

Figure S19. Capacitance corrected cyclic voltammograms at 1600 rpm of a commercial FeNC catalyst, C_2N 900@Fe (cathodic sweep) and Pt/C (anodic sweep).

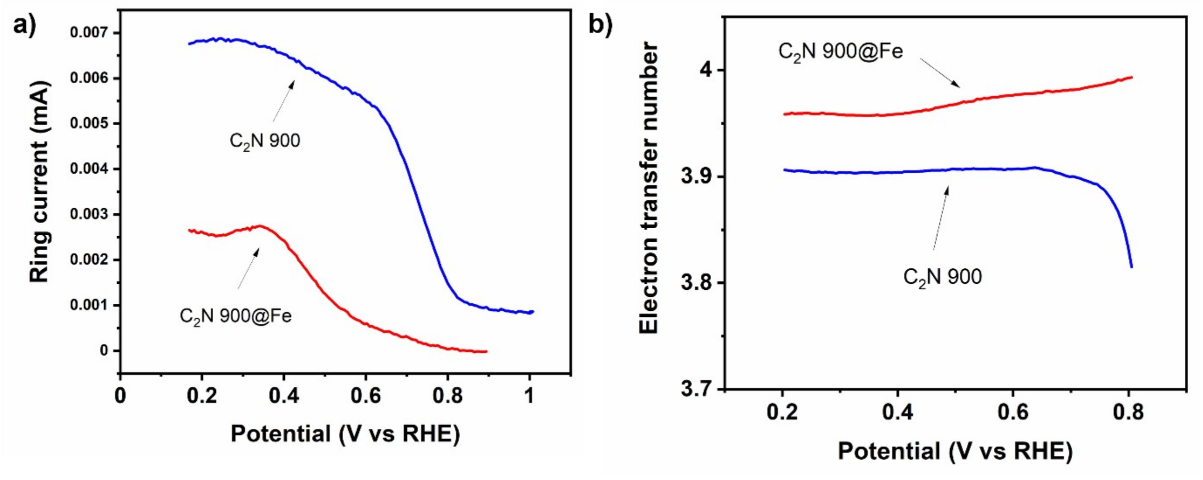

Figure S20. Rotating ring current for the oxidation of hydrogen peroxide in C_2N 900 and C_2N 900@Fe (a) and electron transfer number for C_2N 900 and C_2N 900@Fe

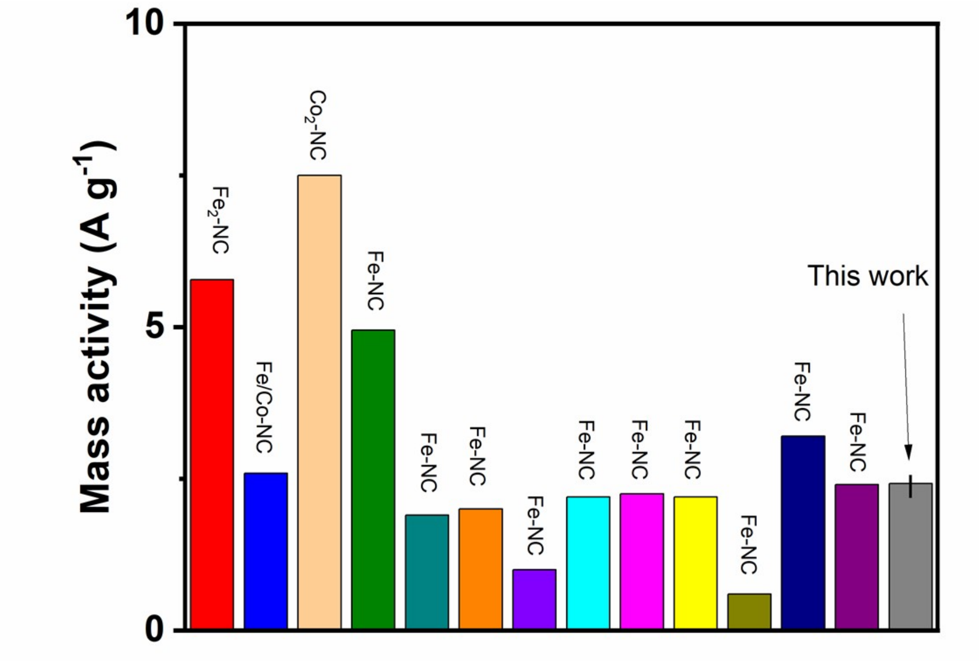

Figure S21. Mass activity of catalysts from the literature at 0.8 V vs RHE. Data reproduced from references 2 (Fe_2-NC, 0.51 mg cm^{-2} 0.5 M H_2SO_4, red), 3 (Fe/Co-NC 1.01 mg cm^{-2} 0.1 M $HClO_4$, blue), 4 (Co_2-NC 1.01 mg cm^{-2} , beige 0.1 M $HClO_4$), 5 (Fe-NC 0.29 mg cm^{-2} 0.1 M $HClO_4$, green and Turquoise), 6 (Fe-NC orange, purple, cyan, magenta, 0.2 mg cm^{-2} 0.5 M H_2SO_4), 6 (yellow, dark yellow, dark blue, violet 0.2 mg cm^{-2} 0.5 M H_2SO_4), C_2N 900@Fe - this work (0.26 mg cm^{-2} 0.1 M $HClO_4$ grey),

Table S4. Comparison of oxygen reduction activity in acidic media between different single and dual atom catalysts from literature.

Catalyst	Type of proposed active site	Electrolyte	Loading (mg cm^{-2})	Half wave potential (V)	Reference
Fe_2/N/C	Dual atom	0.5 M H_2SO_4	0.51	0.78	[2]
Fe_3/N/C	Triple atom	0.5 M H_2SO_4	0.51	0.76	[2]
Fe-Co/N/C	Dual atom	0.1 M $HClO_4$	1.10	0.86	[3]
Fe-CoNx-OH/C	Dual atom	0.1 M $HClO_4$	0.40	0.86	[7]
Zn-Co-Nx/c	Dual atom	0.5 M H_2SO_4	0.31	0.70	[8]
CoPNi-N/C	Dual atom	0.1 M $HClO_4$	0.30	0.73	[9]
Fe/N/C	Single atom	0.1 M $HClO_4$	0.29	0.72	[10]
Fe/N/C	Single atom	0.1 M $HClO_4$	0.29	0.66	[10]
Fe/N/C	Single atom	0.1 M $HClO_4$	0.10	0.66	[11]
Fe/N/C	Single atom	0.5 M H_2SO_4	0.26	0.74	[12]
Fe/N/C	Single atom	0.1 M $HClO_4$	0.60	0.73	[13]
Fe/N/C	Single atom	0.1 M $HClO_4$	0.26	0.73	[14]
**This work**	**Dual atom**	**0.1 M $HClO_4$**	**0.26**	**0.73**	

References

1 Y. Garsany, J. Ge, J. St-Pierre, R. Rocheleau and K. E. Swider-Lyons, *J. Electrochem. Soc.*, 2014, **161**, F628–F640.

2 W. Ye, S. Chen, Y. Lin, L. Yang, S. Chen, X. Zheng, Z. Qi, C. Wang, R. Long, M. Chen, J. Zhu, P. Gao, L. Song, J. Jiang and Y. Xiong, *Chem*, 2019, **5**, 2865–2878.

3 J. Wang, Z. Huang, W. Liu, C. Chang, H. Tang, Z. Li, W. Chen, C. Jia, T. Yao, S. Wei, Y. Wu and Y. Li, *J. Am. Chem. Soc.*, 2017, **139**, 17281–17284.

4 M. Xiao, H. Zhang, Y. Chen, J. Zhu, L. Gao, Z. Jin, J. Ge, Z. Jiang, S. Chen, C. Liu and W. Xing, *Nano Energy*, 2018, **46**, 396–403.

5 D. Menga, J. L. Low, Y.-S. Li, I. Arčon, B. Koyutürk, F. Wagner, F. Ruiz-Zepeda, M. Gaberšček, B. Paulus and T.-P. Fellinger, *J. Am. Chem. Soc.*, 2021, **143**, 18010–18019.

6 M. Primbs, Y. Sun, A. Roy, D. Malko, A. Mehmood, M.-T. Sougrati, P.-Y. Blanchard, G. Granozzi, T. Kosmala, G. Daniel, P. Atanassov, J. Sharman, C. Durante, A. Kucernak, D. Jones, F. Jaouen and P. Strasser, *Energy Environ. Sci.*, 2020, **13**, 2480–2500.

7 M. Xiao, Y. Chen, J. Zhu, H. Zhang, X. Zhao, L. Gao, X. Wang, J. Zhao, J. Ge, Z. Jiang, S. Chen, C. Liu and W. Xing, *J. Am. Chem. Soc.*, 2019, **141**, 17763–17770.

8 D. Liu, B. Wang, H. Li, S. Huang, M. Liu, J. Wang, Q. Wang, J. Zhang and Y. Zhao, *Nano Energy*, 2019, **58**, 277–283.

9 Z. Li, H. He, H. Cao, S. Sun, W. Diao, D. Gao, P. Lu, S. Zhang, Z. Guo, M. Li, R. Liu, D. Ren, C. Liu, Y. Zhang, Z. Yang, J. Jiang and G. Zhang, *Appl. Catal. B Environ.*, 2019, **240**, 112–121.

10 D. Menga, F. Ruiz-Zepeda, L. Moriau, M. Šala, F. Wagner, B. Koyutürk, M. Bele, U. Petek, N. Hodnik, M. Gaberšček and T. Fellinger, *Adv. Energy Mater.*, 2019, **9**, 1902412.

11 L. Lin, Q. Zhu and A.-W. Xu, *J. Am. Chem. Soc.*, 2014, **136**, 11027–11033.

12 Q. Lai, L. Zheng, Y. Liang, J. He, J. Zhao and J. Chen, *ACS Catal.*, 2017, **7**, 1655–1663.

13 A. Kong, X. Zhu, Z. Han, Y. Yu, Y. Zhang, B. Dong and Y. Shan, *ACS Catal.*, 2014, **4**, 1793–1800.

14 F.-L. Meng, Z.-L. Wang, H.-X. Zhong, J. Wang, J.-M. Yan and X.-B. Zhang, *Adv. Mater.*, 2016, **28**, 7948–7955.
